# Supplementary material for: Morphometric based differentiation among Trichogramma spp
Source: PLoS One. 2020 Aug 6;15(8):e0236422. doi: 10.1371/journal.pone.0236422 (PMC7410216; doi:10.1371/journal.pone.0236422)
Supplement: Attachment — Submitted filename: Response to Reviewers.docx [file pone.0236422.s001.docx]

Comments to Reviewer 1:

1. The sentence “In morphometrics, the stressed are mostly on the genitalia characteristics, antennae, forewings of the males for the identification of different species of *Trichogramma*” have been re-written for better clarification.
2. In Introduction, the importance of current work has been added with description.
3. The reason for selecting these five species of *Trichogramma* for morphometric analysis have been added in the material and method section.
4. The material and method section have been reformed with addition on few information such as duration of any treatments and temperature treatment.
5. Figure legend for the numbers are already mentioned in the “Morphometric observation” part of **Material and Methods** section.
6. Figure 1a on page 15 is being attached in separate file as fig 2a for better resolutions. Also, morphometric traits have been incorporated and described in details for each species in their diagnosis section. All morphometrics figures are attached as .tiff file format for better resolution.
7. The Abstract section has been revised by providing some information about the reason for current study, methodology and results.
8. The present study is limited to the morphometric studies based on morphological characters only. Molecular analysis was not carried out in the present study. Molecular is advanced tool but traditional methodology is also a reputed tool for separating different species. In future, I will work on molecular analysis of insects but for now my study is restricted to morphological aspects only.
9. The results and discussion part of the manuscript has been completely revised as per the reviewer comments. The flaws in English language of the manuscript were removed at authors’ best level. The haphazard of the results and discussion part are revised to make a meaningful and understandable results.
10. The conclusion is added in the manuscript as per the reviewer comments.

Comments to Reviewer 2:

1. The objective of the study is added in the Introduction part of the manuscript. Also, the importance of the study is discussed in details.
2. The morphological characters are the basis of present study. The Figure 0 is the demonstration of considered part/character of any body part of either male or female. Also, few characters have been considered for the first time in morphometric analysis. The ratios of few characters were calculated to identify their applicability in morphometric separation of *Trichogramma* spp. So, to avoid confusion among the readers for any abbreviation or characters full name, the Figure 0 is given in the material and methods section. Also, figure 0 will be attached in separate .tiff file as the image is good quality but in MS Word, it become little blur.
3. The morphological diagnosis for all the five species of *Trichogramma* are incorporated in Results and Discussion section.
4. The cultures obtained from the laboratory were identified using the literature available. The details are provided in the Material and Method section.
5. The no. of specimens used for morphometry has been included in the manuscript.
6. The females of *Trichogramma* spp. cultures were identified using the literature available. Hind tibial length and length of aedeagus and apodemes were compared with the ovipositor to identify and confirm the species.
7. As per the earlier researchers, females’ characters are less useful in identification and separation of *Trichogramma* spp. In present study, it was attempted to check whether the female characters could be used for morphological differentiation using morphometry. In this study, few characters of females were found significant in separating studied species. These characters will be highlighted in the manuscript and separate paragraph will be added to show them significantly.
8. References for the terminology used are properly cited in the manuscript. E.g. Yousuf and Shafee, 1988; Hassan and Yousuf, 2007 and Khan *et al*., 2019.
9. All the images of morphometric characters of *Trichogramma* spp. will be uploaded as .tiff file to avoid blurriness.
10. The morphometric observations of the *Trichogramma* spp. were repetitive in table also. So, from observation, repetitive paragraphs are removed. Only characters, which are discussed with previous research work have been shown.
11. The conclusion is added in the manuscript as per the reviewer comments.
12. The results and discussion part of the manuscript has been completely revised as per the reviewer comments. The flaws in English language of the manuscript were removed at authors’ best level. The haphazard of the results and discussion part are revised to make a meaningful and understandable results.
13. In results and discussion part, earlier morphometric observation was given for individual species. After revision, apart from little individual observation on species, a comparative study among the species is also included using the graphs.
