# Supplementary material for: Morphometric based differentiation among Trichogramma spp
Source: PLoS One. 2020 Aug 6;15(8):e0236422. doi: 10.1371/journal.pone.0236422 (PMC7410216; doi:10.1371/journal.pone.0236422)
Supplement: Attachment — Submitted filename: Response to Reviewer.docx [file pone.0236422.s002.docx]

**Comments to Reviewer**

1. The manuscript is edited with the English first language writer who have greatly improved this paper both grammatically and typologically.
2. All the comments suggested by the reviewer are accepted by the author(s). We are thankful to the reviewer for his exhaustive comments on the manuscript which was very essential to make this article a good read.
3. author(s) have also tried to remove all flaws in the manuscript and modified the manuscript as per the reviewer guidelines.
4. As per the reviewer comments in Introduction part, sentence “In the natural system, the insect population is being regulated by various factors like temperature, sunlight crop composition and others” has been Omitted and sentence is re-formed.
5. As reviewer suggested about the elaboration and more indication on “shows positive correlation (≥0.200)”, we have mentioned about the intensity of correlation. Also, detailed description has been added in the manuscript about the statistics behind these PCs (Principal Components) and characters association. These correlations are related with different principal components but not with the species itself. As there is large no. of variables so all the variable could not participate in attaining greater association with PCs. Though, some of these characters all together can generate good correlation and this is the objective of the study to finalize and identify the group of characters which can helpful in identification and separation of the species in *Trichogramma*.
6. In Conclusion part, reviewer commented on “So, that a document can be prepared to identify the correct species for better use in biological control programme”. The authors appreciated the query and information has been added to the manuscript about the type of document and its content. The sentence has been revised as So that a reference-document consisting of protocol, guidelines, species-based information, morphometric data, key and others can be prepared to identify the correct species for better use in biological control programme.
7. The reviewer comments have been taken into consideration. Venkatesan *et al*. (2016) in Biological Control (doi: 10.1016/j.biocontrol.2016.07.005) is actually, Thiruvengadam *et al*. (2016). The authors have compared the molecular taxonomy work by Thiruvengadam *et al*. (2016) with the morphological taxonomy work of present study.
8. The author agrees with the reviewer suggestion regarding the molecular taxonomy of *Trichogramma* spp. The present study is limited to the identification based on morphology only. In future studies, we will start working on molecular aspect too. Molecular is the latest technological methodology being utilized widely in insect taxonomy. Still, most of the insect taxonomies around the world are based on morphological characters only. These characters are vital in the separation of the different species of any genus. Specifically, in *Trichogramma* spp. morphological characters such as genitalia length, width, ovipositor length, forewings characteristics are often utilized for its identification by various researchers such as Nagaraja and Nagarkatti (1969), Garcia-Gonzalez; Rohi and Pintureau, 2003; Hernandez-Gonzalez (2009) and Querino et al. (2017). As the taxonomic inferences described by morphology are more reliable than any other method. Even, most of the researchers who have maximum no. of species under their credit are still depends on morphological based taxonomy. Few researchers such as Ranyse B. Querino, Roberto A. Zucchi, J.D. Pinto, Nagaraja and Nagarkatti have primarily chosen morphological based identification in most of their studies.

So, in future studies, the molecular technique will be employed along with the morphological methods but for now, we are limited with morphological character-based identification due to various reason.

Thanking you

.
